# The Reliability and Quality of Videos as Guidance for Gastrointestinal Endoscopy: Cross-Sectional Study

**DOI:** 10.2196/58855

**Published:** 2025-03-11

**Authors:** Jinpei Liu, Yifan Qiu, Yilong Liu, Wenping Xu, Weichen Ning, Peimei Shi, Zongli Yuan, Fang Wang, Yihai Shi

**Affiliations:** 1 Department of Gastroenterology Gongli Hospital of Shanghai Pudong New Area Shanghai China; 2 College of Basic Medicine Sciences Second Military Medical University/Naval Medical University Shanghai China; 3 Department of Gastroenterology Changzheng Hospital Naval Medical University Shanghai China; 4 Department of Respiratory and Critical Care Medicine Changzheng Hospital Naval Medical University Shanghai China

**Keywords:** gastrointestinal endoscopy, YouTube, patient education, social media gastrointestinal, large language model, LLM, reliability, quality, video, cross-sectional study, endoscopy-related videos, health information, endoscopy, gastroscopy, colonoscopy

## Abstract

**Background:**

Gastrointestinal endoscopy represents a useful tool for the diagnosis and treatment of gastrointestinal diseases. Video platforms for spreading endoscopy-related knowledge may help patients understand the pros and cons of endoscopy on the premise of ensuring accuracy. However, videos with misinformation may lead to adverse consequences.

**Objective:**

This study aims to evaluate the quality of gastrointestinal endoscopy-related videos on YouTube and to assess whether large language models (LLMs) can help patients obtain information from videos more efficiently.

**Methods:**

We collected information from YouTube videos about 3 commonly used gastrointestinal endoscopes (gastroscopy, colonoscopy, and capsule endoscopy) and assessed their quality (rated by the modified DISCERN Tool, mDISCERN), reliability (rated by the *Journal of the American Medical Association*), and recommendation (rated by the Global Quality Score). We tasked LLM with summarizing the video content and assessed it from 3 perspectives: accuracy, completeness, and readability.

**Results:**

A total of 167 videos were included. According to the indicated scoring, the quality, reliability, and recommendation of the 3 gastrointestinal endoscopy-related videos on YouTube were overall unsatisfactory, and the quality of the videos released by patients was particularly poor. Capsule endoscopy yielded a significantly lower Global Quality Score than did gastroscopy and colonoscopy. LLM-based summaries yielded accuracy scores of 4 (IQR 4-5), completeness scores of 4 (IQR 4-5), and readability scores of 2 (IQR 1-2).

**Conclusions:**

The quality of gastrointestinal endoscope-related videos currently on YouTube is poor. Moreover, additional regulatory and improvement strategies are needed in the future. LLM may be helpful in generalizing video-related information, but there is still room for improvement in its ability.

## Introduction

Gastrointestinal endoscopy is a medical procedure performed by the insertion of an endoscope into the digestive tract through the oral and anal routes. It allows direct visualization of the inner lining of the esophagus, stomach, and intestines and enables the treatment of various conditions, such as ulcers, polyps, and tumors [1]. In recent years, the incidence and gradual rejuvenation of gastrointestinal diseases have increased [2]. As a significant screening and diagnostic tool for gastrointestinal diseases, gastrointestinal endoscopy is increasing in importance. Gastroscopy and colonoscopy screening have significantly increased the rate of early diagnosis of gastrointestinal cancers, reduced mortality, and improved disease prognosis [3,4]. However, previous studies have shown that 38.1% of people refuse to undergo gastroscopy because they do not sufficiently recognize the knowledge related to this examination [5]. More seriously, some patients are delayed in the diagnosis and treatment of their disease because of misinformation about gastroscopy [6]. Capsule endoscopy is another important gastrointestinal examination technique that has emerged over the past few years. It has the advantages of being painless, noninvasive, and relatively less demanding to operate [7]. However, due to insufficient knowledge of the indications, contraindications, and effects of capsule endoscopy [8], it is difficult for patients to independently make a choice that is appropriate for their condition. Therefore, providing medical education to patients about the indications, contraindications, and key points of preoperative preparation for various gastrointestinal endoscopic techniques can help them receive appropriate treatment in a timely manner and may improve the outcome of their disease.

In recent years, the increasing popularity of mobile electronic devices and the development of internet applications have led to a rising utilization of social media in the medical field [9,10]. Social media platforms that disseminate health information web-based have been proven to enhance the efficiency of doctors' consultations and improve the experience of patients [11-13]. Due to the convenience of accessing information, an increasing number of patients are turning to social media platforms as a source of health-related information [11]. In the field of gastroenterology, social media-based telemedicine is actively involved in patient health care for patients and in doctors’ decision-making [12]. It has been reported that communication through social media improves the quality of bowel preparation for patients undergoing colonoscopy [13]. Notably, the advent of large language models, such as ChatGPT (OpenAI), has facilitated faster access to endoscopic information [14]. Interestingly, a video content summarization tool built on the foundation of big language models offers a new option for individuals who are unable or unwilling to watch an entire video [15].

YouTube has emerged as a paramount platform for disseminating web-based content, boasting an impressive global user base of 256 million [16]. Moreover, the amount of medical content on this platform has increased significantly in recent years [17]. Used appropriately, platforms such as YouTube can prompt behavioral change in viewers, enhancing their health [18]. However, YouTube also allows the distribution of unverified and nonprofessional content [19], leading to an increase in low-quality videos, which are created by nonspecialist users with little to no medical training [6]. This compromises the overall quality of medical content on YouTube, providing potentially misleading health information to unsuspecting viewers. Therefore, additional efforts from health care professionals are needed to correct patients' misconceptions from the abovementioned misinformation during their treatments, reducing the efficiency of health care services and potentially escalating doctor-patient conflicts [20,21]. Currently, a substantial number of gastrointestinal endoscopy-related videos have emerged on YouTube. However, there is a lack of research evaluating the quality of these videos.

The aim of the present study was to examine the quality, reliability, and recommendability of videos related to gastroscopy, colonoscopy, and capsule endoscopy available on YouTube. Additionally, we also sought to assess the practicality of a large language model (LLM)–based video content summarization tool for summarizing health-related video content [15].

## Methods

### Search Strategy and Selection Approach for the Included Videos

**Figure 1:**
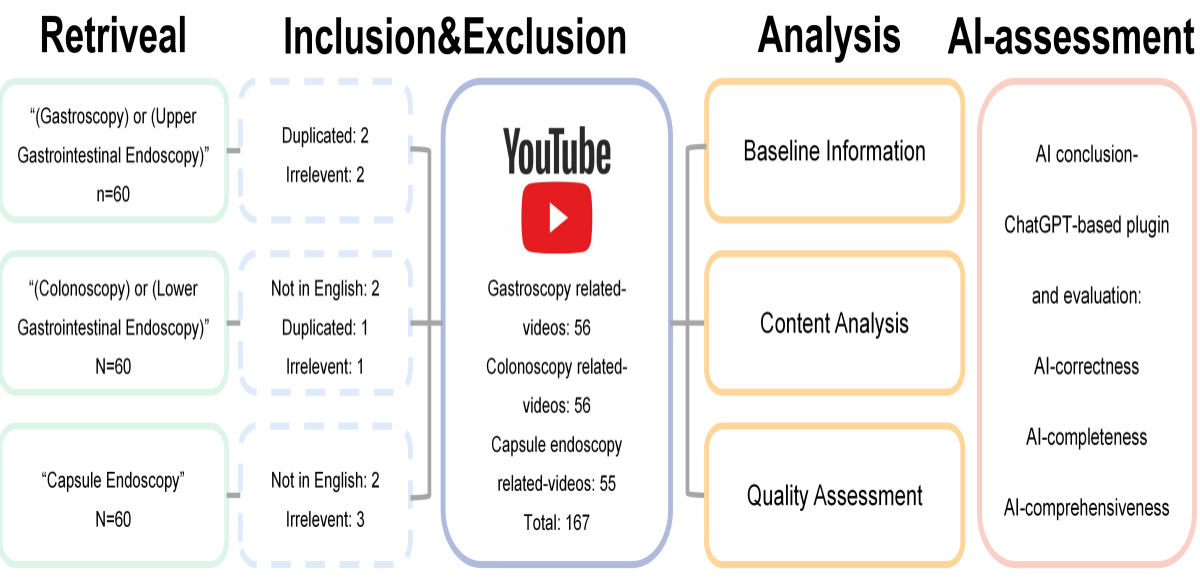
Search strategy, video screening procedure, and flowchart of this research. AI: artificial intelligence.

All the videos included in the analysis were obtained from the web-based YouTube platform. The keywords used were “(gastroscopy) or (upper gastrointestinal endoscopy),” “(colonoscopy) or (lower gastrointestinal endoscopy),” and “capsule endoscopy.” To minimize the impact of the personalized recommendation algorithm on the inclusion of videos, we registered 3 separate brand-new accounts to retrieve the 3 different endoscopies. No filter settings were used during the retrieval process to faithfully simulate the usage scenarios of patients or other regular users. The search process for videos of the 3 gastrointestinal endoscopies was completed between September 28, 2023, and September 30, 2023. Duplicate videos, non-English videos, and videos that were not relevant to the search topic were excluded. Based on the experience from previous studies and the browsing habits of video platform users, namely, they mostly prioritize top search results over extensive browsing on video platforms [22-27], we selected videos that appeared within the top 5 pages of the YouTube interface, which is a total of 60 results for each endoscope for subsequent analysis. The specific retrieval and filtering process is shown in Figure 1.

### Evaluating Methodologies of the Videos

The reliability and content quality of the videos were evaluated using the Modified DISCERN (mDISCERN), the *Journal of the American Medical Association* (*JAMA*) score, and the Global Quality Scoring (GQS) scale. Before this assessment, the participating physicians familiarized themselves with the endoscopy guidelines and official descriptions of the 3 scales, enabling them to assess the accuracy and completeness of the video content while adhering to the guidelines. The mDISCERN scale was initially devised by Charnock et al [28] for evaluating written health information and was employed to examine video content quality [29]. The scale's modified version contains a 5-question questionnaire (Table S1 in the Multimedia Appendix), providing 1 point for each affirmative response for a maximum of 5 points. Videos scoring 3 or more on the mDISCERN scale were deemed high-quality health information providers [29]. The *JAMA* Rating Scale (Table S2 in the Multimedia Appendix) was used to assess health-related video information reliability [30]. It includes 4 assessment criteria: authorship, attribution, disclosure, and timeliness, with each carrying a 1-point score of up to a total of 4. Higher *JAMA* scores suggest better reliability of health information [30]. The GQS (Table S3 in the Multimedia Appendix) uses a 1-5 scale across 5 criteria, with greater GQS indicating a more highly recommended video [31]. Additionally, the artificial intelligence (AI)–summarized content was evaluated in terms of accuracy, reliability, and readability (Table S4 in the Multimedia Appendix), with accuracy-related elements divided into 6 dimensions from 1 to 6 and completeness and readability divided into 3 dimensions from 1 to 3.

### Data Collection and Evaluation of Video Reliability and Content Quality

Essential video information was gathered for subsequent analysis, including video length, release date, number of days since release, play count, likes, steps, comments, uploader's follower count, uploader's identity, and the Video Power Index (VPI). We divided the uploaders into 4 groups based on their status: doctors or hospitals, medical media, other health organizations (including medical associations, medical examination centers, equipment companies, etc), and patients. The VPI, used to measure video popularity, was computed with the formula VPI=([number of likes]/[number of likes+number of dislikes])×100)×([number of views/days after upload]/100) [32]. Additionally, the ratios of (number of likes+number of steps)/plays and number of comments/plays were calculated to evaluate viewer interaction levels. The LLM processes the raw text extracted from video content, including both text and subtitles, and subsequently synthesizes a summary and general insights. To avoid sequence bias, the video order was randomized. All videos included in the analysis and the content synthesized by LLM were assessed by 2 senior gastroenterologists (YL and YF). For scoring items prompting disagreement, a third senior physician (WP) made the final decision.

### Statistical Analysis

The nonnormal distribution of the entire data set was initially confirmed using the Shapiro-Wilk test. The median, interquartile range, mean, and variance were calculated for the continuous variables and the frequency of the categorical variables. For continuous variables that deviated from a normal distribution, the Wilcoxon-Mann-Whitney test and the Kruskal-Wallis test were used for between-group differences. All the data analyses were carried out with IBM SPSS Statistics for Mac (version 27; IBM Corp) via a 2-sided test approach. A *P* value of less than 0.05 was considered to indicate statistical significance. Moreover, for comparisons among multiple subgroups, Bonferroni corrections were applied to the *P* values. Finally, data visualization was performed using R software (R Core Team, the R Foundation).

## Results

### Video Features

**Figure 2:**
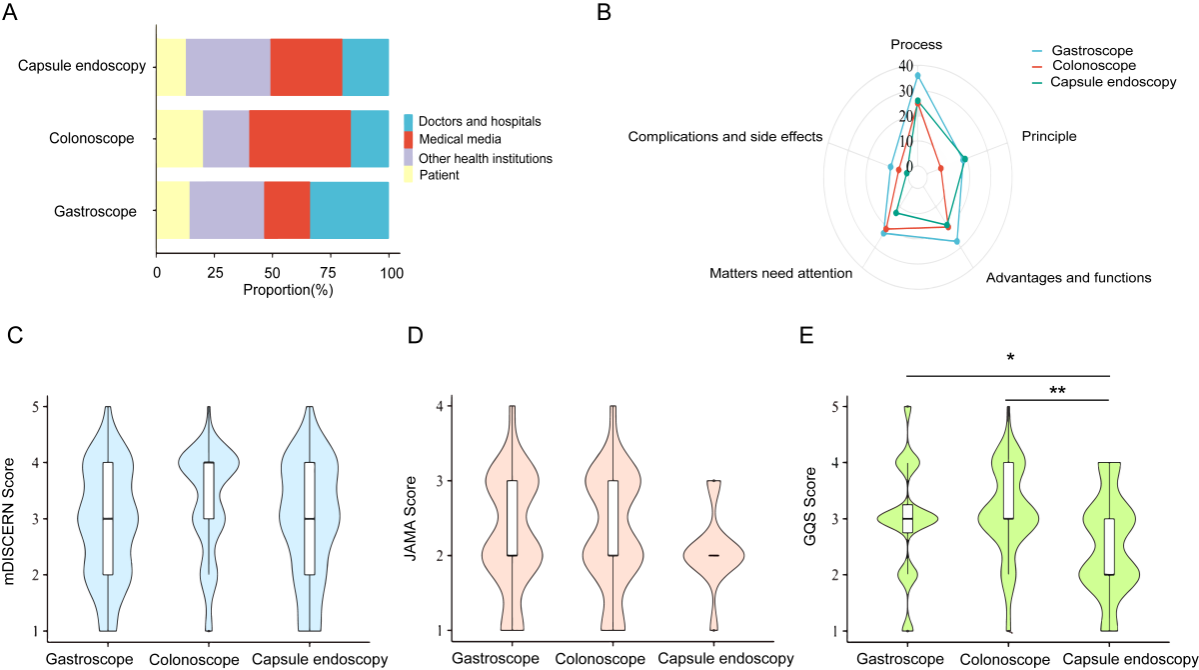
The characteristics and quality of gastrointestinal-related videos on YouTube. (A) Proportion of the 4 groups of the uploaders. (B) Radar charts showing the percentage of each kind of content categorization among videos of different types of endoscopes. (C) mDISCERN score, (D) JAMA score, (E) GQS score of videos of different types of endoscopes. * denotes *P*<.05, and ** denotes *P*<.01 using the Kruskal-Wallis test. GQS: Global Quality Score; JAMA: Journal of the American Medical Association.

A total of 167 relevant videos were included in the analysis; 56 were gastroscopy-related videos, 56 were colonoscopy-related videos, and 55 were capsule endoscopy-related videos. We divided the uploaders into 4 groups based on their status: doctors or hospitals, medical media, other health organizations (including medical associations, medical examination centers, equipment companies, etc), and patients (Figure 2A). Most of the videos were uploaded via platforms with medically related backgrounds, including medical media (n=52, 31.3%), other health organizations (n=49, 29.5%), and doctors or hospitals (n=39, 23.5%; Figure 2A). Colonoscopy-related videos were relatively longer, with a median of 288 (IQR 159-477) seconds, followed by gastroscopy-related videos (median 251, IQR 115-530) and capsule endoscopy-related videos (median 213, IQR 110-487). Capsule endoscopy-related videos were significantly less popular (median VPI 4.45, IQR 1.75-15.59) than gastroscopy-related (median VPI 32.47, IQR 7.77-88.20) and colonoscopy-related (median VPI 31.52, IQR 9.69-129.52) videos were. The trend in the number of views was also similar to that for the VPI; gastroscopy-related videos and colonoscopy-related videos both had significantly more plays than did capsule endoscopy-related videos, while there was no significant difference between the former two (Table 1).

**Table 1:** Baseline characteristics of videos about gastroscopy, colonoscopy, and capsule endoscopy.

Video features	Gastroscopy			Colonoscopy			Capsule endoscopy	
	Median (IQR)	Mean (SD)	Median (IQR)		Mean (SD)	Median (IQR)		Mean (SD)
VPI^a^	32.47 (7.77-88.2)^b^	267.11 (1318.94)	31.52 (9.69-129.52)^b^		400.16 (1231.56)	4.45 (1.75-15.59)		9.92 (11.90)
Days after upload	1810 (844-3937)	2288.23 (1681.80)	2024 (968-3279)		2213.11 (1410.74)	2225 (697-3442)		2270.27 (1553.14)
Number of views	74,402 (7967-211,364)^b^	402,609.09 (1,244,985.57)	56,967 (21,073-197,575)^b^		776,618.05 (2,775,980.36)	10,003 (1690-22,691)		23,024.84 (33,509.27)
Video length	251 (115-530)	350.41 (314.71)	288 (159-477)		404.41 (426.41)	213 (110-487)		322.35 (284.35)
Number of likes	301 (60-1128)	901.38 (1403.43)	376 (99-1653)		4951.29 (16,833.17)	26 (6-111)		86.93 (128.95)
Number of dislikes	18 (1-65)	75.04 (125.28)	18 (3-60)		236.63 (769.59)	0 (0-5)		4.78 (8.41)
Subscribers of the uploaders	16,900 (1970-130,000)	284,096.68 (1,085,446.40)	48,400 (8580-20,5000)		340,198.88 (1,022,888.32)	3090 (661-23,800)		25,285.58 (48,619.33)
Number of comments	35 (1-126)	96.94 (171.78)	35 (2-202)		350.65 (1137.62)	1 (0-20)		12.91 (22.56)
Number of likes+dislikes or views	49.07 (27.87-83.96)	87.49 (157.30)	62.69 (33.11-122.41)		94.84 (91.92)	44.09 (24.81-79.58)		83.54 (157.11)
Number of comments or views	1.51 (0-10.96)	13.20 (30.21)	1.21 (0-14.89)		13.81 (28.80)	0.66 (0-3.78)		34.41 (173.05)

^a^VPI: Video Power Index.

^b^Significantly different from capsule endoscopy-related videos; *P*<.05 using the Kruskal-Wallis test.

### Video Content

Based on the guidelines and the videos included in the analysis, we categorized the content of the 3 types of endoscopy videos. We assessed the comprehensiveness of the included videos’ content from the following five perspectives: (1) process: this refers to the process by which a patient undergoes gastrointestinal endoscopy; (2) principle: this refers to the principles of construction and operation of gastrointestinal endoscopes; (3) advantages and benefits: this refers to the roles of gastrointestinal endoscopy techniques, indications, and roles in relation to other investigations; (4) matters that need attention: this means that the patient needs to be aware of the following things before, during, and after the gastrointestinal endoscopic examination; and (5) complications and side effects: possible complications and adverse effects of endoscopic techniques in the digestive system. As shown by the radar chart (Figure 2B), among the 3 types of videos, the content coverage of gastroscopy-related videos was broadest, and the coverage of colonoscopy-related videos and capsule endoscopy-related videos was approximate; both of these videos were narrower than that of gastroscopy-related videos. Of the 167 endoscopy-associated videos analyzed, a substantial majority encompassed depictions of the process and enumerated potential advantages or benefits, accounting for 87 (52.1%) and 66 (39.5%) videos, respectively. In stark contrast, only 15 (9.0%) videos addressed possible complications and adverse reactions associated with gastrointestinal endoscopy.

### Information Quality and Reliability

We evaluated the quality and reliability of the videos across different endoscope types and within groups of the same endoscope type (Table 2). On average, colonoscopy-related videos had an mDISCERN of 3.29 (SD 0.94), which was slightly greater than that of both gastroscopy (2.88, SD 0.99) and capsule endoscopy (2.93, SD 1.05), albeit not significantly (Figure 2C). In contrast, capsule endoscopy-related videos significantly underperformed, with a GQS of 1.88 (SD 0.83) when compared to gastroscopy (2.96, SD 0.97; *P*=.03) and colonoscopy (3.13, SD 0.79; *P*<.01; Figure 2D). However, the *JAMA* scores across all 3 endoscopy types remained similar (Figure 2E).

**Table 2:** mDISCERN, JAMA^a^, and GQS^b^ scores of the gastroscopy, colonoscopy, and capsule endoscopy.

Category	mDISCERN			*JAMA*			GQS	
	Median (IQR)	Mean (SD)	Median (IQR)		Mean (SD)	Median (IQR)		Mean (SD)
Gastroscopy	3 (2-4)	2.88 (0.99)	2 (2-3)		2.29 (0.78)	3 (2-4)		2.96 (0.97)
Colonoscopy	4 (3-4)	3.29 (0.94)	2 (2-3)		2.29 (0.79)	3 (3-4)		3.13 (0.79)
Capsule endoscopy	3 (2-4)	2.93 (1.05)	2 (2-2)		2.07 (0.50)	2 (2-3)		2.53 (0.88)

^a^JAMA: Journal of the American Medical Association.

^b^GQS: Global Quality Scoring.

**Figure 3:**
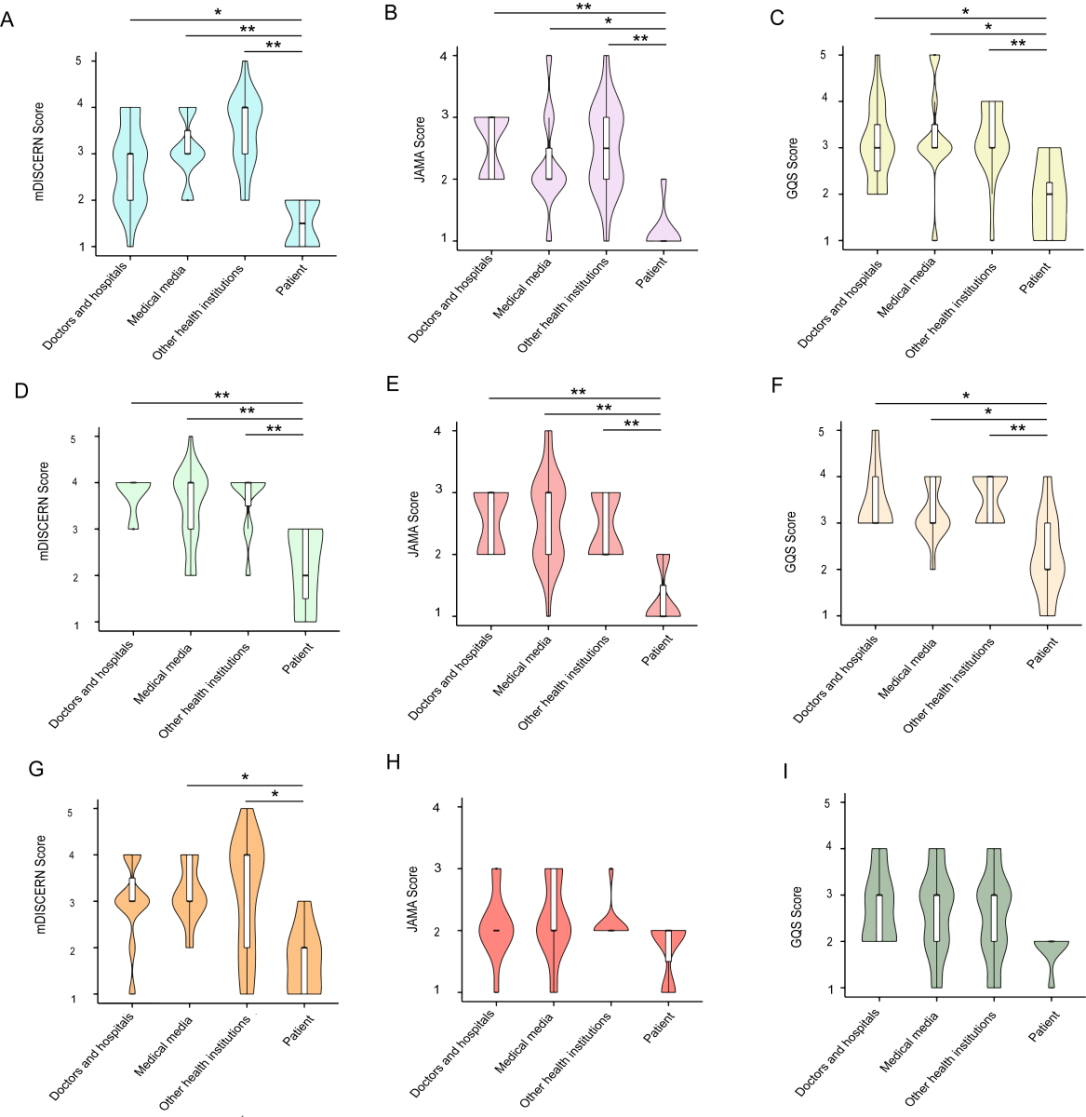
The reliability and quality of gastrointestinal endoscopy from different identities of the uploaders. (A) mDISCERN score, (B) JAMA score, and (C) GQS score of videos about gastroscopy from different identities of the uploaders. (D) mDISCERN score, (E) JAMA score, and (F) GQS score of videos about colonoscopy from different identities of the uploaders. (G) mDISCERN score, (H) JAMA score, and (I) GQS score of videos about capsule endoscopy from different identities of the uploaders. * denotes *P*<.05, and ** denotes *P*<.01 using the Kruskal-Wallis test. GQS: Global Quality Score; JAMA: Journal of the American Medical Association.

In terms of the different sources of the video uploaders (Table 3), we observed that the GQS scores of patient-provided gastroscopy-related videos were significantly lower than those of doctors or hospitals (1.88, SD 0.83 vs 3.05, SD 0.85; *P*=.04), medical media (1.88, SD 0.83 vs 3.27, SD 1.10; *P*<.01), and other professional organizations (1.88, SD 0.83 vs 3.45, SD 0.52; *P*=.02; Figure 3C). Consistent with the GQS results, the mDISCERN score (1.50, SD 0.53) and the *JAMA* score (1.13, SD 0.35) were also lower for patient-provided gastroscopy-related videos than for the other 3 videos (Figures 3A and B). Similarly, when analyzing colonoscopy-related videos, we found that the scores of patient-provided videos were lower than those of the other 3 videos in all 3 categories (Figures 3D-F). Interestingly, while patient-provided capsule endoscopy-related videos were scored lower in quality than videos provided by medical media (mDISCERN: 1.71, SD 0.76 vs 3.18, SD 0.64; *P*<.05) and other professional organizations (mDISCERN: 1.71, SD 0.76 vs 3.10, SD 1.25; *P*<.05), there was no significant difference in quality between patient-provided videos and videos provided by doctors or hospitals according to all the mDISCERN scores, *JAMA* scores and GQS scores (Figures 3G-I).

**Table 3:** mDISCERN, JAMA^a^, and GQS^b^ scores of videos from 4 groups of the uploaders.

Source			Videos, n		mDISCERN					*JAMA*					GQS		
					Median (IQR)		Mean (SD)		Median (IQR)			Mean (SD)		Median (IQR)			Mean (SD)
**Gastroscopy**																	
	Doctors or hospitals	19		3 (2-3)		2.74 (0.87)		3 (2-3)			2.58 (0.51)		3 (2-4)			3.05 (0.85)	
	Medical media	11		2 (2-3)		3.09 (0.70)		2 (2-3)			2.27 (0.79)		3 (3-4)			3.27 (1.10)	
	Other health institutions	18		4 (3-4)		3.50 (0.79)		3 (2-3)			2.50 (0.71)		3 (3-4)			3.45 (0.52)	
	Patients	8		2 (1-2)		1.50 (0.53)		1 (1-1)			1.13 (0.35)		2 (1-3)			1.88 (0.83)	
**Colonoscopy**																	
	Doctors or hospitals	9		4 (4-4)		3.78 (0.44)		3 (2-3)			2.56 (0.53)		3 (3-4)			3.44 (0.73)	
	Medical media	24		4 (3-4)		3.46 (0.83)		3 (2-3)			2.58 (0.72)		3 (3-4)			3.21 (0.59)	
	Other health institutions	11		4 (3-4)		3.64 (0.67)		2 (2-3)			2.45 (0.52)		4 (3-4)			3.55 (0.52)	
	Patients	11		2 (1-3)		2.18 (0.87)		1 (1-2)			1.27 (0.47)		2 (2-3)			2.27 (0.90)	
**Capsule endoscopy**																	
	Doctors or hospitals	11		3 (3-4)		3.00 (0.89)		2 (2-2)			2.09 (0.54)		3 (2-3)			2.73 (0.79)	
	Medical media	17		3 (3-4)		3.18 (0.64)		2 (2-3)			2.18 (0.64)		3 (2-3)			2.65 (0.93)	
	Other health institutions	20		4 (2-4)		3.10 (1.25)		2 (2-2)			2.10 (0.31)		3 (2-3)			2.55 (0.94)	
	Patients	7		2 (1-2)		1.71 (0.76)		2 (1-2)			1.71 (0.49)		2 (2-2)			1.86 (0.38)	

^a^JAMA: Journal of the American Medical Association.

^b^GQS: Global Quality Scoring.

### Conclusion and Evaluation

**Figure 4:**
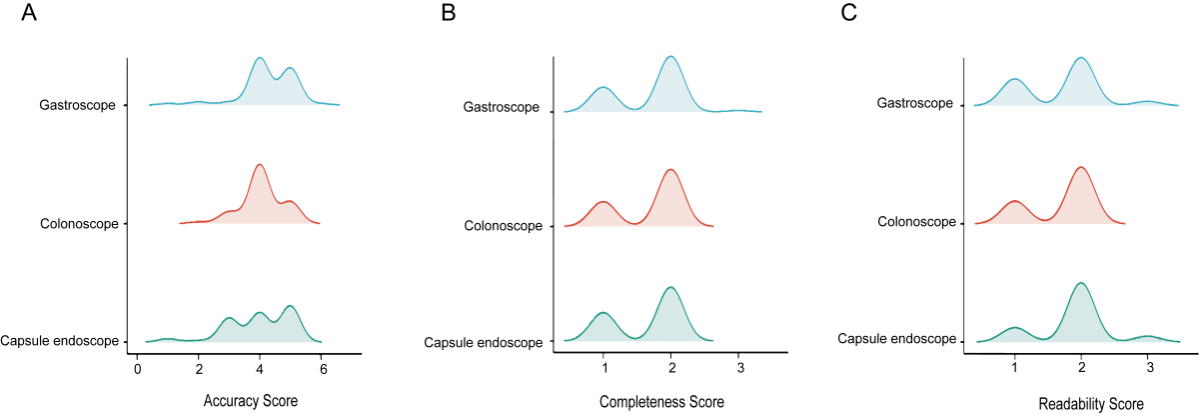
AI-conclusion and evaluation of the videos. (A) Accuracy score, (B) completeness score, and (C) readability score among different types of endoscopes. AI: artificial intelligence.

To assess the utility of the LLM-based video content summarization tool for summarizing health videos related to gastrointestinal endoscopy, we imported the 167 videos and collected the content summarized by the video content summarization tool. The summarized content was then evaluated in terms of accuracy, completeness, and readability (Figures 4A-C). LLM-based summaries yielded accuracy scores of 4 (4-5), completeness scores of 2 (1-2), and readability scores of 2 (1-2). None of the 3 scores were significantly different among the 3 gastrointestinal endoscopies.

## Discussion

### Major Findings

In this study, we reviewed the relevant videos about gastroscopy, colonoscopy, and capsule endoscopy posted on YouTube before October 2023. First, we collected basic information such as the number of plays, posting time, number of likes, number of dislikes, and VPI of these videos and categorized and summarized the video content in 5 aspects. Second, we evaluated the quality, reliability, and degree to which the related videos were recommended. Finally, we explored the value of the LLM-based video summarization tool for health-related videos.

Previous research has indicated that mDISCERN scores and GQS scores less than 3 often indicate lower quality and lower levels of recommendation [19]. In our study, the average mDISCERN score was 2.88 for gastroscopy-related videos and 2.93 for capsule endoscopy-related videos. Only 38.9% (67/167) of the videos were assigned an mDISCERN score higher than 3, and for GQS scores, the proportion of videos with a score higher than 3 was even lower. Moreover, only 23.4% (39/167) of the videos were assigned a value above 3 when considering both criteria. Therefore, the overall quality and recommendability of these videos were insufficient. A surprising conclusion is that even professionally produced videos are not always suitable for viewer recommendations. This phenomenon may be attributed to 3 potential reasons: first, a small number of videos were too specialized and not applicable to patients; second, some videos were broad and lacked in-depth information; and third, certain videos were outdated, had lower production quality, and offered a poor viewing experience. In the present study, the mean *JAMA* score for both gastroscopy- and colonoscopy-related videos was 2.29, while for capsule endoscopy-related videos, it was only 2.07. These results suggest that the reliability of gastrointestinal endoscopy videos currently available on YouTube is also questionable. Specifically, we discovered that the majority of video producers did not include references or additional sources of information. Furthermore, some physicians featured in the videos were hesitant to disclose their affiliation unless they were promoting a specific institution. These results are similar to those of previous studies in that some funded physician users did not explicitly disclose conflicts of interest in their YouTube videos [33]. This lack of transparency compromises the videos' reliability and adds to viewer distress. Fortunately, although many videos lacked sufficient information, we did not find any videos that provided clearly incorrect information. When assessing content, our radar chart analysis revealed that only a small number of videos mentioned the potential complications and side effects of endoscopy in the digestive system.

A previous study revealed that the quality of health-related videos varies among authors with different backgrounds [34]. Specifically, in the context of gastrointestinal endoscopy videos, except for the *JAMA* scores of the capsule endoscopy group, videos created by individuals with a medical specialty background demonstrated higher levels of quality and reliability than those produced by nonmedical users. While there was no significant discrepancy in the *JAMA* scores for capsule endoscopy between medical professionals and patients, videos created by professionals still scored higher on average. This outcome can be attributed to 2 potential reasons. First, professionals possess greater familiarity with clinical guidelines and the latest research findings, whereas nonprofessionals are more inclined to share personal experiences and lessons learned. Second, medical professionals are skilled at presenting relevant information coherently and in an organized manner, while videos produced by laypeople tend to be more relaxed and casual, which, to some extent, affects patient quality [35].

### Video Education Is an Important Part of Patient Education

Health education plays a pivotal role in patient care [36]. Providing high-quality health education to patients not only enhances patient-physician collaboration and facilitates smoother treatment processes but also contributes to a partial reduction in complications and alleviates the health care burden on society [37]. A previous study demonstrated that physician-led telephone education improved patient preparation prior to gastrointestinal endoscopy, thus enhancing the detection of lesions in the digestive system [38].

With the increasing popularity of short video sharing platforms, increasingly more health-related short videos are becoming known to the public [39]. Compared to traditional text-based information, videos are more interesting and intuitive [40]. High-quality videos play a crucial role in enhancing patients' understanding of the disease and increasing the likelihood of their acceptance of treatment plans [41]. Additionally, they effectively alleviate patient anxiety and enhance satisfaction levels [42]. Therefore, the role of video platforms will become increasingly important in patient education.

### The Use of AI in Health-Related Information Dissemination Is Debatable

AI has played a significant role in identifying fake health-related information, as evidenced by studies on the detection of COVID-19 misinformation through machine learning algorithms [43,44]. However, there is a lack of research on AI applications for content summarization and information extraction in health-related videos. The video summarization tool used in this study is based on the LLM model, which summarizes video content using the text and subtitles present within the video [15]. This subtitle-based AI video content summarization tool not only offers a quick summary of the video content but also strives to maintain consistency with the original video. Nonetheless, this approach has several limitations. Upon comparing the text generated by the LLM-based video summarization tool with the actual video content, we observed instances where the tool missed key points and overly emphasized minor details. Moreover, providing a summary solely based on subtitles means that the LLM tool does not truly “watch” the video or refine its content, potentially leading to the omission of valuable information provided by the video creators beyond the subtitles, thereby reducing the comprehensiveness of the summary. The computer graphics and live-action scenes provided in videos allow viewers to create visual impressions, a dimension that the LLM tool lacks in the summarization process. Significantly, the present study indicates that for videos that lack subtitles and rely solely on image presentations, the LLM summarization tool tends to rely on the video title, resulting in discrepancies in accuracy and completeness when compared to the actual video content. Therefore, while summarizing health-related videos using existing LLM-based video summarization tools can enhance viewers' information absorption efficiency to some degree, the nature of this approach suggests that it cannot truly serve as a substitute for the experience of watching a video, which enables viewers to form a more intuitive understanding of diseases and treatment techniques.

### Limitations and Future Directions

This study has several limitations. First, only English videos posted on the YouTube platform were analyzed, making it challenging to directly generalize the findings to other platforms or videos in different languages. Second, the video content summarization plugin used in this study was developed based on ChatGPT (version 3.5), and a plugin based on ChatGPT (version 4.0) may yield improved performance.

Many current gastrointestinal endoscopy videos lack details on equipment makers and resolutions. Though not crucial for patient preparation, such information can help viewers better understand the equipment used during procedures. We suggest future videos include this data consistently. We recognize the absence of mandatory policies governing health education videos [40]. As video-sharing platforms continue to gain popularity, it becomes crucial to implement a certain level of censorship and regulation for videos disseminated on the internet. Government agencies and video-sharing platforms should establish a body composed of health care professionals dedicated to screening and monitoring health-related videos uploaded web-based and promptly identifying and flagging videos containing controversial information. Given the intricacy of medical content, we recommend that individuals with professional backgrounds in health care exercise caution when using specialized vocabulary when producing videos and strike a balance between popularity and the scientific nature of educational videos. This approach ensures that a broader audience can comprehend the information presented in the videos. Additionally, labeling the sources of arguments and information in the video will enhance its reliability and further improve viewers' acceptance of the content.

### Conclusions

This study evaluated the information quality of videos discussing gastroscopy, colonoscopy, and capsule endoscopy on the YouTube platform. Overall, the videos exhibited an average relatively low quality. Notably, videos created by professionals had a higher level of reliability and quality than did those produced by nonprofessionals. We look forward to the availability of a greater number of high-quality gastroenterology-related videos in the future, as they greatly contribute to providing patients with valuable health-related information. Additionally, we urge the government to swiftly implement policies that curtail the dissemination of low-quality health-related videos.

## Abbreviations

AI: artificial intelligence
GQS: Global Quality Score
JAMA: Journal of the American Medical Association
LLM: large language model
VPI: Video Power Index

## Data Availability

The data that support the finding of this study are openly available.
